# Team Coping: Cross-Level Influence of Team Member Coping Activities on Individual Burnout

**DOI:** 10.3389/fpsyg.2021.711981

**Published:** 2021-11-04

**Authors:** Wim Kamphuis, Roos Delahaij, Thomas A. de Vries

**Affiliations:** ^1^Department of Human Behaviour and Training, Netherlands Organisation for Applied Scientific Research (TNO), Soesterberg, Netherlands; ^2^Department of Human Resource Management & Organisational Behaviour, University of Groningen, Groningen, Netherlands

**Keywords:** coping, burnout, military, multilevel theory, stress, teams, resources, resilience

## Abstract

Coping with stress has been primarily investigated as an individual-level phenomenon. In work settings, however, an individual’s exposure to demands is often shared with co-workers, and the process of dealing with these demands takes place in the interaction with them. Coping, therefore, may be conceptualized as a multilevel construct. This paper introduces the team coping concept and shows that including coping as a higher-level team property may help explain individual-level outcomes. Specifically, we investigated the effects of exposure to danger during deployment on burnout symptoms in military service members and examined to what extent this relationship was moderated by individual-level and team-level functional coping. We hypothesized that the relationship between individuals’ exposure to danger and burnout is contingent on both. In line with our predictions, we found that service members who were highly exposed to danger, and did not engage in much functional coping, suffered most from burnout symptoms, but only when their teammates did not engage in much functional coping either. When their teammates did engage in much functional coping, the effect of exposure to danger on burnout was buffered. Hence, team members’ coping efforts functioned as a resilience resource for these service members.

## Introduction

Some jobs place extremely high demands on employees. Police officers, paramedics, firefighters, and military personnel, for example, are frequently exposed to life-threatening situations and hostile working conditions (Skogstad et al., 2013; Coenen and van der Molen, 2021). To prevent such exposure to danger from damaging psychological well-being and mental health (e.g., Pietrzak et al., 2009; Ramchand et al., 2015), individuals in highly demanding jobs need to engage in coping (Jex et al., 2001). Coping has been defined as “cognitive and behavioral efforts to manage specific external and/or internal demands that are appraised as taxing or exceeding the resources of the person” (Lazarus and Folkman, 1984, p. 141). Effective coping has been shown to moderate the effects of acute and chronic job demands on health, work engagement, and burnout outcomes (De Rijk et al., 1998; Angelo and Chambel, 2014; Searle and Lee, 2014).

Although much is known about how individual coping may moderate the stressor–strain relationship, far less is known about how this process is influenced by the social environment in which this takes place. Importantly, however, today’s work often takes place in teams where individuals work closely together towards joint outcomes (Ilgen et al., 2005). Police officers, firefighters, and paramedics, for example, typically work as closely knit subgroups of emergency response teams, whereas military personnel often operate in cohesive battle squads (Goodwin et al., 2012). In such team settings, individuals are generally exposed to the same life-threatening situations and, consequently, cope with these demands by interacting with each other, exchanging experiences, and providing mutual support. As such, individual team members’ coping strategies may emerge into a collective phenomenon within teams. Although it seems plausible that such team coping can substitute or reinforce individual members’ coping, research on this topic is largely missing.

**Figure 1:**
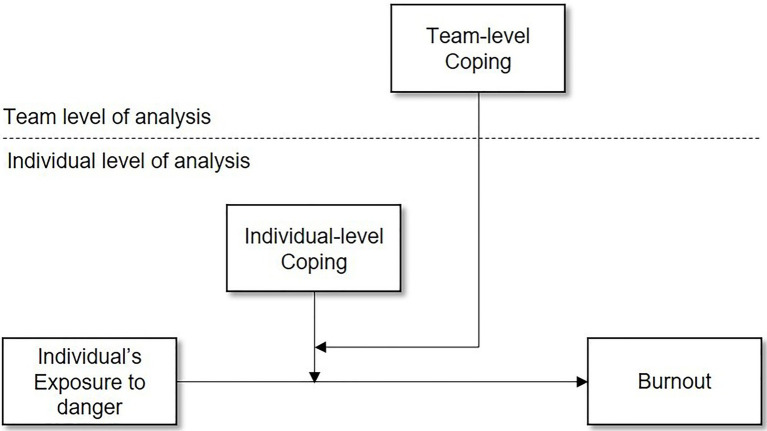
Conceptual multilevel model of coping.

The aim of the present study is to address this issue and examine the effects of “team coping” (i.e., the combined efforts made by members of a team to manage work demands exceeding their resources, that influences individual team members’ outcomes) on individual well-being. To this end, the present study uses a multilevel approach to investigate coping in team settings, including coping as both an individual level phenomenon and a team level property (cf. Kozlowski and Klein, 2000). Team coping will be especially relevant for work settings where members of teams are confronted with the same job demands and work closely together in dealing with these demands. This study therefore focuses on military teams during deployment, investigating to what extent coping at the individual and team level influence the relationship between danger encountered during the mission and symptoms of burnout [a response to work-related stressors, defined by exhaustion, cynicism, and inefficacy (Maslach et al., 2001)] of service members part of this mission. We expect that the relationship between exposure to danger and symptoms of burnout will be influenced by both individual-level and team-level coping, such that the influence of individual-level coping depends on the degree of team-level coping (see Figure 1).

Investigating coping as a team-level property has both theoretical and practical relevance. Most research in this area has treated the appraisal and coping process as a purely individual undertaking. By directing attention to the coping strategies used by the immediate social environment of the individual, this traditional view of coping is extended, opening up new directions in coping research. Knowledge of the effects of team coping may help better support professionals confronted with stressful situations as part of their job. Organizations could use this knowledge to design work processes in such a way that individuals are not only facilitated to deal with these demands individually, but also collectively, as a team. By mobilizing the coping capabilities of coworkers, professionals in high-risk occupations may be better equipped to perform well in stressful situations, and stay healthier and motivated in the long term.

### Coping

Before we elaborate on the team coping concept, we first briefly introduce individual-level coping research, with a focus on the current perspective of what constitutes functional coping. According to the transactional model of stress and coping (Lazarus and Folkman, 1984), stress results from the appraisal of an imbalance between demands and resources, and coping consists of cognitive and behavioral strategies aimed at restoring the balance. Different types of coping strategies have been identified. A widely used typology distinguishes problem-focused strategies from emotion-focused strategies. The former entail strategies aimed at managing the stressor, while the latter entail strategies aimed at managing the distress caused by the stressor (Folkman and Moskowitz, 2004). This perspective has clarified that a person needs a broad pallet of both problem-focused and emotion-focused strategies to effectively deal with different kinds of stressors. Specifically, individuals need problem-focused strategies to effectively deal with stressors in controllable situations (e.g., Cohen et al., 2008), while they need emotion-focused strategies in more uncontrollable situations (e.g., Britt et al., 2016). Riolli and Savicki (2010), for example, showed that among US service members, those who applied a diverse set of problem-focused and emotion-focused coping strategies showed better psychological adjustment after traumatic stress. As such, a combination of both problem- and emotion-focused coping strategies can be deemed functional (i.e., leading to adaptive short term and long term outcomes) for coping with stress in high-risk settings (Aldwin, 2007).

What is still missing in current coping research, however, is a detailed consideration of an individual’s social surrounding on his or her coping effectiveness. This is an important omission, considering that individuals typically do not work in isolation, but instead closely collaborate with each other in order to realize common, team-level goals. In such interdependent social settings, the process of appraising and dealing with these demands will not be confined to isolated individuals, but rather take place in the interaction between them (Hobfoll, 2001). Consequently, through individuals interacting about and coping with these common demands, a higher-level shared “team coping” construct may emerge. This higher level team coping may in turn influence lower-level outcomes, such as an individual’s work engagement and burnout, either directly, or indirectly by shaping or moderating relationships and processes at the lower level (Kozlowski and Klein, 2000).

### Team Coping

Consider a project team that has to perform in an extremely demanding environment, with high stakes and tight deadlines. At a certain moment, one of the team members falls ill, and the remaining members are left with an almost impossible task. This may give rise to a large amount of stress, and may eventually lead to burnout symptoms in the remaining team members. The way these team members individually cope with this situation determines whether they will suffer these negative outcomes or not; if an individual does not engage in functional coping, the outcomes may be impaired performance, reduced health and lowered well-being. But because the individual members are part of a team, the coping behavior of the other team members may matter as well: One member, for example, may use humor to deal with the situation, helping the others to put things in perspective. Or another member may use his social skills to get help from someone outside the project, relieving the pressure on the remaining project members. In this manner, the combined coping efforts by members of the team to manage work demands exceeding the team’s resources and may have benefits for the individual team members, over and above the effects of their own coping efforts.

Although coping has been investigated in teams, coping literature does not encompass a clear multilevel conceptualization of coping. Previous research on coping in teams can be broadly divided into two lines of research. The first line focuses on the coping strategies and personality characteristics of individual team members that face significant stressors or extreme conditions in different work domains (e.g., Leon et al., 1989; Burns et al., 2008). This line of research, however, does not address how other team members’ coping strategies influence a focal person’s coping outcomes. The second line of research addresses the effects of stress on teams and the strategies employed by these teams to cope with team task demands (e.g., Driskell et al., 1999; Pearsall et al., 2009; Kamphuis, 2010; Kamphuis et al., 2011). This line of research has primarily focused on how team performance can be protected against negative effects of stress. Outcome measures in these studies therefore typically relate to the team level (e.g., team cohesion, team performance). This line of research, however, does not consider team-level coping’s influence on individual-level outcomes. Just as individual-level coping research largely neglected the influence of an individual’s social surrounding on his or her coping effectiveness, team-level coping research ignored the effects of team member coping efforts on individual-level outcomes.

With a prior consideration of multilevel effects of coping lacking, in the present study, we examine cross-level relationships between team coping and individual’s coping on the one hand, and individual team members’ wellbeing on the other hand. Team coping originates from team members’ individual efforts to cope with common demands. These individual efforts may differ from each other and may not converge to a common coping strategy. Some team members, for example, may use problem-focused coping strategies in response to a specific demand, whereas other team members may use more emotion-focused coping strategies, and still others may not use functional coping strategies at all. Despite these differences, the individual coping strategies all contribute to the team’s collective dealing with the common demand, regardless of whether there is an agreement between team members in the use of coping strategies, and whether all team members employ coping strategies equally. As noted before, one team member’s humor, for example, may also help other team members to put a situation into perspective and cope with stressful events. In sum, the individual contributions to team coping may or may not be isomorphic or converge among members (Kozlowski and Klein, 2000), and instead may vary in amount and type depending on individuals idiosyncratic characteristics (e.g., personality, cognitions, and attitudes). We therefore follow an “additive model” to explain how team coping emerges from individual members’ efforts (Chan, 1998; Kozlowski and Klein, 2000) and operationalize team coping as “a summation of the lower level units (i.e., members’ coping) regardless of the variance among these units” (Chan, 1998, p. 236).

Team coping, in turn, may influence the individual team members’ coping outcomes. The coping strategies used by team members may change the focal individual’s appraisal of the situation, which in turn may influence his or her outcomes. During a stressful situation, there is a constant interaction between primary appraisal (is the situation positive, negative, or irrelevant?) and secondary appraisal (can something be done about the situation?), which determines the severity and nature of stress reactions experienced (Lazarus and Folkman, 1984; Lazarus, 1999). When a team member is confronted with a serious demand, the coping strategies used by other members may influence both types of appraisals for the focal person. One’s team members’ coping strategies may make the situation seem less threatening, for example, because team members use humor as a coping strategy. It can also make the situation seem more manageable, for example, because team members use problem-focused strategies to solve the situation. In both instances, the demanding situation is expected to cause less stress, and thus to result in less negative outcomes for the individual. Building on these insights, we suggest that the team coping exhibited by a person’s teammates may determine the need, and thus the effectiveness, of the respective person’s individual coping.

### Present Study: Demands and Outcomes in a Military Context

In the present study, we investigated coping in a military context. Previous research has identified a number of job demands characteristic of military operations. These demands include powerlessness, isolation, boredom, and danger (Bartone et al., 1998; Boermans et al., 2013). The current study focused on danger as the key demand. Danger encompasses threats to the safety of oneself or one’s comrades. These threats include combat situations, attacks (e.g., improvised explosive devices, missiles, or suicide attacks), and accidents.

Exposure to such high job demands might lead to depletion of energy and disengagement from work, and eventually result in burnout when insufficient coping resources can be deployed (Bakker et al., 2005). Burnout can be described as a work-related syndrome that may develop in response to occupational stressors in various occupational settings (Demerouti et al., 2001; Chirico, 2016). Prolonged exposure to work-related demands without sufficient resources to cope with these demands may lead to chronic exhaustion and cynicism (Bakker et al., 2014). Research has shown that this process may also take place in a military context (e.g., Chappelle et al., 2019; Elliman et al., 2021; Gottschall and Guérin, 2021). Service members in deployment situations, who are exposed to danger and trauma in war, run the risk of developing burnout symptoms when insufficient coping takes place (Vinokur et al., 2011; Delahaij et al., 2016). By using effective coping strategies, however, these negative effects may be buffered, and energy and engagement may be preserved (Bakker et al., 2005). In this study, we therefore investigate to what extent the exposure to danger causes symptoms of burnout in military service members during their deployment, and how coping moderates this relationship.

We examine whether or not individual-level and team-level functional coping jointly moderate the relationship between an individual members’ exposure to danger and his or her burnout in a military setting. As described above, the effectiveness of individual coping strategies depends on the type of demands encountered (Lazarus and Folkman, 1984). A dangerous encounter in the military typically consists of both controllable and uncontrollable components. During the encounter, problem-focused strategies will be effective in neutralizing the threat (Delahaij et al., 2011). After the encounter, being able to process psychological distress, for example by seeking social support, is important (Pietrzak et al., 2010). As such, strategies aimed at actively dealing with the encounter, as well as strategies aimed at dealing with the uncontrollable aspects of the encounter, and the emotions that may follow the encounter, can be deemed functional for coping with the exposure to danger during deployment. Hence, at the individual level, there will be a positive relationship between exposure to danger and burnout symptoms when the individual’s use of functional coping strategies is low. When, on the other hand, the individual’s use of functional coping strategies is high, the effect of the exposure to danger will be buffered, such that the relationship between exposure to danger and burnout will weaken, or diminish.

However, we propose that the relationship between exposure to danger, individual-level coping and burnout will also be influenced by team-level coping, since team members share the exposure to danger and interact in coping with it. Specifically, we expect that team-level coping may be particularly useful for members who do not use many functional coping strategies themselves. For these individuals, exposure to danger is likely to cause high stress, both during the encounter, and afterwards. In these instances, the use of functional coping strategies used by other team members may be beneficial, because team members’ coping strategies may change the focal individual’s appraisal of the situation, such that the situation is perceived as less threatening and/or more manageable, resulting in a less stressful experience for the individual. For team members who already use functional coping strategies themselves, we do not expect an additional effect of team coping, because these members already successfully buffer the effect of the exposure to danger on burnout themselves. Specifically, we hypothesize (see Figure 1):

1. Burnout symptoms of soldiers are influenced by a three-way interaction between exposure to danger, individual coping, and team coping.
  - Exposure to danger has a stronger, more positive relationship with burnout symptoms when the use of functional coping strategies at both the individual level and the team level is low.
  - Exposure to danger has a weaker, less positive relationship with burnout symptoms when the use of functional coping strategies at either the individual level or the team level is high, or when coping at both levels is high.

## Materials and Methods

### Participants and Procedure

The present study was conducted with service members of Netherlands Armed Forces (NLDAF) who were either deployed in the NATO mission ISAF in Afghanistan or the NATO anti-piracy mission in the Gulf of Aden. The sample included service members from the Police Training Group and the Air Task Force within the ISAF mission and two rotations of the Anti-Piracy mission. Cross-sectional data were collected as part of the standardized leadership and mental health support assessment conducted by the Behavioral Sciences Institute of the NLDAF. The study was approved by the institute’s Ethical Review Board. Participation was voluntary and anonymity assured. 686 participants, distributed across 50 teams, returned a completed survey. Because the survey is part of the leadership and mental health support during deployment, involvement of service members is high, and the typical response rate is around 90%. Three teams were excluded from the analyses because the number of participants was too low to be considered a team (<3 respondents) and one team (of 67 respondents) was excluded because it was suspected that this team in reality consisted of multiple separate teams which had accidently received the same identifier in the analyses. The teams differed in nature (e.g., battle units, technical units, logistical units, and staff) and in typical size. The teams in our sample ranged from 3 to 37 respondents (M=13.35; SD=8.78). Respondents were mostly male (94%), held non-officer ranks (97%), and were on average 32.71years of age (SD=9.95).

### Measures

#### Exposure to Danger

Congruent with prior research (Bartone et al., 1998), we measured individuals’ exposure to danger by asking respondent how often they had (1) engaged in combat activities, (2) been under attack, and (3) witnessed accidents on a seven-point scale (0 “never” to 6 “continuously”). We then calculated individuals’ exposure to danger as their average response on these three items. Cronbach’s Alpha was 0.84.

#### Individual Coping

We measured individuals’ functional coping during deployment using the COPE questionnaire (Carver et al., 1989). Because data collection took place in a high-pressure operational environment, restrictions existed with regard to the number of items that could be administered in the survey. We therefore used a short form of the original COPE questionnaire, created for the NLDAF (Delahaij et al., 2014). This short form consists of a scale of 8 items belonging to the strategies deemed most functional for service members of the NLDAF during deployments (i.e., active coping, positive reinterpretation, seeking social support, acceptance, and humor). Respondents were asked to what extent they had used these coping strategies to deal with stressful situations they encountered during the mission on a 5-point scale, ranging from 1 “never” to 5 “continuously.” We treated coping as a formative construct, in which the use of different functional strategies may all contribute to overall functional coping. We were not interested in the effects of separate strategies, but rather in the multilevel effects of the functional coping repertoire as a whole. We therefore computed individuals’ coping as their average response to all eight items. A higher score indicates more use of different functional coping strategies. Cronbach’s Alpha was 0.65, which is comparable to other studies using similar individual-level coping measures (e.g., Carver, 1997; Aldwin, 2007).

#### Team Coping

As noted above, team coping originates from team members’ individual efforts to cope with common demands. Although these individual efforts all serve to deal with common (team-level) demands, individual team members may differ from each other and may not converge to a common coping strategy because they may have different routines or preferences for dealing with demands. Correspondingly, we followed an additive model logic (Chan, 1998) and averaged individual team members’ coping scores to obtain a measure for the degree to which their team, as a whole, engaged in coping. To assess the appropriateness of the additive model, we calculated the degree to which team members converged in their levels of coping by calculating intraclass correlation coefficient (ICC) statistics. We found minimal convergence between team members’ coping scores (ICC[1]=0.01, n.s.; ICC[2]=0.05, see Table 1), which is coherent with our additive model logic for the team-level coping construct (Chan, 1998).

**Table 1:** Means, standard deviations, and correlations of the study variables.

Individual-level variables	*M*	*SD*	1	2	3		
1. Isolation	1.24	0.89					
2. Exposure to danger	0.38	0.65	0.31^**^				
3. Individual coping	3.17	0.49	−0.09^*^	−0.00			
4. Burnout	1.23	0.94	0.34^**^	0.22^**^	−0.06		
Team-level variables	*M*	*SD*	*ICC1*	*ICC2*	1	2	3
1. Team size	13.35	8.78	-	-			
2. Exposure to danger	0.35	0.21	0.02	0.21	0.20		
3. Team coping	3.19	0.15	0.01	0.05	−0.15	−0.19	
4. Burnout team average	1.13	0.40	0.14^*^	0.67^*^	0.40^**^	0.37^**^	0.01

*N*=517 to 607 individuals for individual-level variables and *N*=46 for team-level variables.

* *p*<0.05;

** *p*<0.01.

#### Burnout

Burnout was measured using a version of the Maslach Burnout Inventory—General Survey (Schaufeli et al., 1996) adapted for the Dutch military (Van Boxmeer et al., 2010). This specific version focuses on the exhaustion and cynicism dimensions of burnout, which are combined in one scale consisting of 8 items. Respondents were, for example, asked to indicate how often they “questioned the usefulness of their work,” “felt emotionally drained,” and “felt burned out from work” on a 7-point scale, ranging from 0 “never” to 6 “continuously.” Cronbach’s alpha value was 0.90.

#### Covariates

We considered team size a control variable, as members of smaller teams may have more opportunity to interact and support each other in their coping. We obtained information on teams’ size from objective records. In addition, we considered respondents’ feelings of isolation and boredom as covariates that may affect their levels of coping and burnout (Bartone et al., 1998). Specifically, based on Bartone et al. (1998), we measured isolation and boredom by asking respondents how often they (1) had felt separated, (2) had to work with unfamiliar persons, (3) had to execute routine work in a repetitive manner, and (4) had little to do for extended time periods. This scale used a 7-point scale format, ranging from 0 “never” to 6 “continuously.” Cronbach’s alpha value was 0.72.

### Analysis

We tested for our cross-level interaction hypothesis following the steps outlined by Aguinis et al. (2013). First, we centered individual-level predictor and moderator variables (e.g., exposure to danger, individual coping) using the group mean, and we centered our team-level variables (e.g., team coping, team size) using the grand mean. Subsequently, we ran four multilevel models to examine our predictions. In the first step, we examined a null model that only included a random intercept for burnout (see Model 1, Table 2). In the next model, we added study covariates, main effects, and all possible two-way interactions between predictor variables (see Model 2, Table 2). In the third step, we assessed whether the strength of the relationship between exposure to danger, individual coping, and burnout varied across teams. To do so, we added the random slope for the interaction coefficient exposure to danger × individual coping (τ12), as well as an estimate for the covariance between the random slope and random intercept (τ01) in Model 3, Table 2. In the final step, we examined our hypothesized three-way interaction effect by regressing the random slope of the two-way interaction term exposure to danger × individual coping on team coping (see Model 4, Table 2). We calculated the R-square increase between the tested model and the null model (i.e., the model including only a random intercept) using Snijders and Bosker's (1999) corrected formula for calculating level-1 R-square measures.

**Table 2:** Regression coefficients from multilevel regression predicting burnout from exposure to danger (ED), individual-level coping (IC), and team-level coping (TC).

Parameter	Null (Model 1)	Random intercept and fixed slope (Model 2)	Random intercept and random slope (Model 3)	Three-way cross-level interaction (Model 4)
Level 1 (L1)				
Intercept (ϒ_00_)	1.16	1.27	1.28	1.28
Isolation		0.41^**^ (0.05)	0.41^**^ (0.05)	0.41^**^ (0.05)
ED (ϒ_10_)		0.15^**^ (0.05)	0.17^**^ (0.06)	0.18^**^ (0.05)
IC (ϒ_11_)		−0.02 (0.05)	−0.04 (0.06)	−0.05 (0.06)
ED×IC (ϒ_12_)		−0.06 (0.12)	−0.06 (0.15)	−0.03 (0.10)
Level 2 (L2)				
Team size		0.01^†^ (0.01)	0.01^*^ (0.01)	0.01^†^ (0.01)
TC		0.10 (0.40)	−0.03 (0.43)	0.01 (0.39)
Cross-level interactions				
ED×TC		0.33 (0.31)	0.23 (0.24)	0.12 (0.24)
IC×TC		0.10 (0.33)	0.17 (0.35)	0.38 (0.36)
IC×TC×ED				1.61^*^ (0.66)
Variance components				
Within-team (L1) variance (σ^2^)	0.77	0.61	0.61	0.61
Intercept (L2) variance (τ_00_)	0.10	0.08	0.08	0.08
Slope (L2) variance (τ_10_)		0.00	0.00	0.00
Slope (L2) variance (τ_11_)		0.00	0.00	0.00
Slope (L2) variance (τ_12_)			0.06	0.02
Intercept-slope (L2) covariance (τ_01_)			0.06	0.03
Additional information				
ICC	0.12			
−2 log likelihood (FIML)	1623.11	1250.83	1249.92	1246.63^†^
No of estimated parameters	3	13	15	16
Pseudo *R*^2^ (Level 1)	0	0.205	0.209	0.214

FIML=Full information maximum likelihood estimation; *N*=516 and L2 sample size=46. Values in parentheses are standard errors; t-statistics were computed as the ratio of each regression coefficient divided by its standard error.

† *p*<0.10.

* *p*<0.05;

** *p*<0.01.

## Results

### Descriptive Statistics

Means, standard deviations, and bivariate correlations for all variables are reported in Table 1. As expected, exposure to danger was positively associated with individuals’ burnout (*r*=0.22, *p*<0.01). Also, both isolation (*r*=0.41, *p*<0.01) and team size (*r*=0.40, *p*<0.01) were significantly related to burnout. This illustrates the relevance of considering these variables as covariates in the present study (cf. Becker, 2005). These correlations should be interpreted with caution, however, given the nested data structure.

### Hypothesis Testing

**Figure 2:**
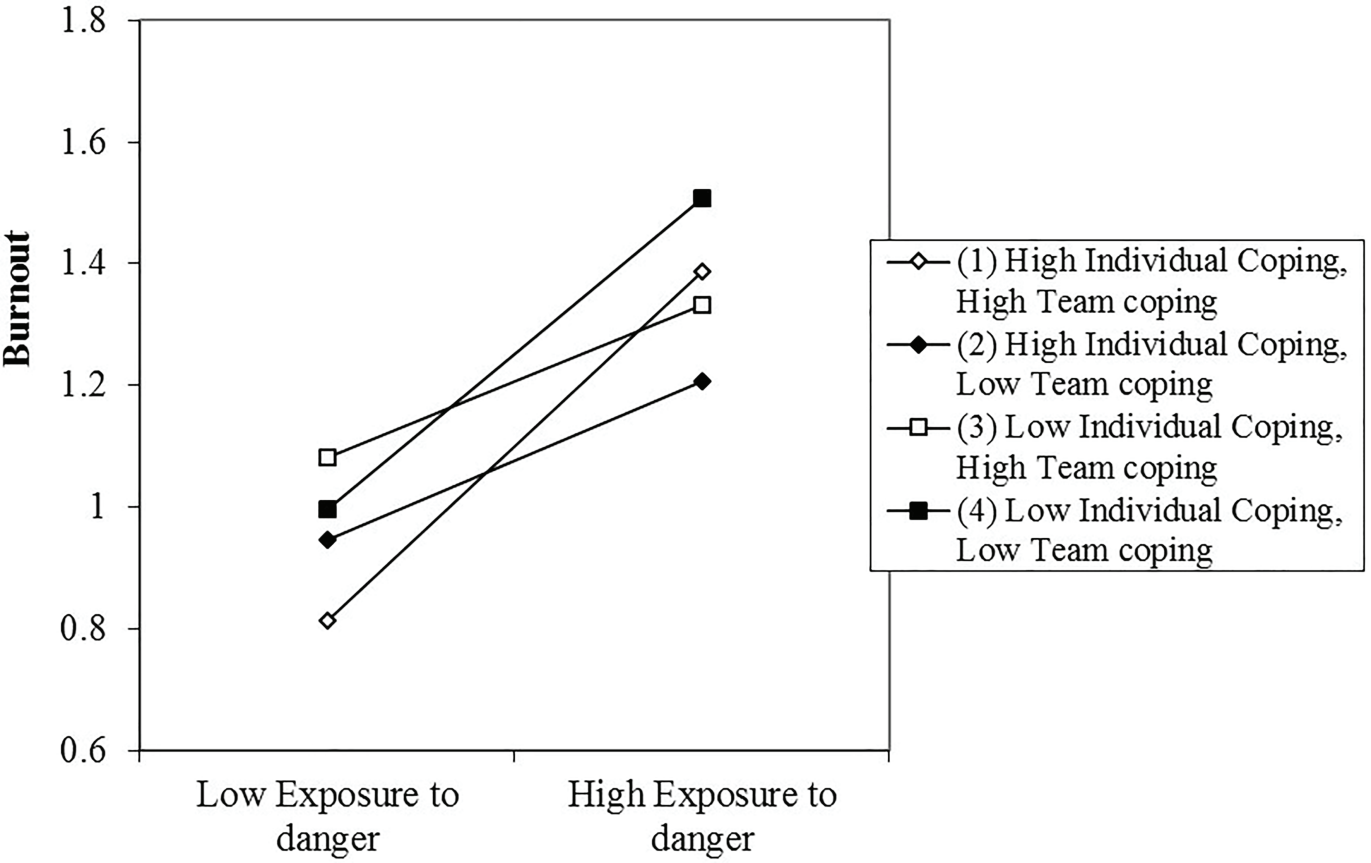
Interactive relation of exposure to danger, individual-level coping, and team-level coping with burnout.

Our hypothesis predicted that the relationship between individuals’ exposure to danger and burnout is contingent on individual-level and team-level coping. The positive relationship between individuals’ exposure to danger and burnout should be accentuated when the use of functional coping strategies at both the individual level and the team level is low (Hypothesis 1a), and attenuated when the use of functional coping strategies at either the individual level or the team level, or both levels is high (Hypothesis 1b). We examined this hypothesis by regressing the random slope of the two-way interaction term Exposure to Danger×Individual Coping on Team Coping (Aguinis et al., 2013). After controlling for covariates, main effects, and all possible two-way interactions between our predictor variables, we found a significant three-way interaction as hypothesized (B=1.61, SE=0.66, *p*<0.05; see Table 2, Model 4). In a subsequent step, we plotted the three-way interaction (see Figure 2) and tested the significance of the simple slopes at different combinations of higher (+1.0SD) and lower (−1.0SD) values of our moderator variables (Cohen et al., 2003). In line with Hypothesis 1a, we found a significant positive relationship between exposure to danger and burnout when individual coping and team coping are both low (B=0.30, SE=0.13, *p*<0.05). In line with Hypothesis 1b, this relationship turns nonsignificant when team coping is high and individual coping is low (B=0.09, SE=0.06, n.s.), and when individual coping is high and team coping is low (B=0.03, SE=0.07, n.s.). Unexpectedly, we also found a positive relationship between exposure to danger and burnout when individual and team coping are both high (B=0.30, SE=0.09, *p*<0.05). We interpret these findings in the discussion section.

## Discussion

The current study introduces the concept of team coping as a team-level property that influences individual-level outcomes in the stress process. The purpose of this study was to investigate the added value of a multilevel conceptualization of coping by determining the cross-level influence of team coping on individual-level burnout outcomes. In doing so, we aimed to extend the coping literature, in which coping is almost exclusively treated as an individual-level phenomenon. Adding the social context, especially in work settings that rely heavily on team work, may contribute to better understanding the coping process and eventually help better support employees in dealing with stress.

The results of our field study with Dutch soldiers deployed on NATO missions show that coping at the team level does indeed contribute to predicting individual level outcomes, over and above the influence of individual-level coping. A three-way cross-level moderation was found in which team coping moderated the effects of individual coping on the relationship between exposure to danger and burnout symptoms. This result shows that it has value to treat coping as a multilevel construct, and that including the team level in models of coping may add to the explanation of individual-level outcomes.

Specifically, in line with our hypothesis, we found a positive relationship between exposure to danger and burnout symptoms when there was limited use of functional coping strategies by the individual and his or her teammates. Also in line with our expectations, this relationship disappeared when the individual’s use of functional coping strategies was high, showing a buffering effect of the functional coping strategies on the relation between danger and burnout. But most interestingly, we found that the harmful influence of exposure to danger could also be buffered by team coping: Service members who were highly exposed to danger during their deployment, and who did not engage in much functional coping themselves, benefited from the coping efforts of their teammates, such that when their teammates did engage in functional coping, exposure to danger did not result in more burnout symptoms. As such, in line with our hypothesis, team coping appeared to function as a compensatory mechanism for lack of individual coping.

Finally, we expected no additional effect of team coping for service members who already use functional coping strategies themselves, because these individuals already successfully buffer the effect of the exposure to danger on burnout themselves. We indeed did not find this effect, however, we did not expect that when individual coping and team coping are both high, the buffering effect of coping would disappear altogether. Nonetheless, this is what the results show. Based on the data collected in our study, we cannot explain this finding. A potential explanation, that deserves future research, however, is that in teams showing this pattern, there were conflicts, or otherwise problematic team processes, resulting in a higher burnout score and potentially also a higher need for coping by each of the members of the team. Since the measurement of team conflicts was beyond the scope of this study, we can only suggest this as a potential explanation. Future research is necessary to further investigate this unexpected result.

### Theoretical Implications

These findings advance coping theory and provide directions for new research. The transactional model of stress (Lazarus and Folkman, 1984) focuses on the appraisal and coping process. The majority of research in this area has directed attention at the role of personal appraisal and the question of which personal coping strategies are effective to restore the balance (e.g., Carver et al., 1989; Day and Livingstone, 2001; Riolli and Savicki, 2010). The present research shows that the effectiveness of the coping process does not only depend on the coping strategies used by the person itself, but also on the coping strategies used by the immediate social environment of the person. This extends the traditional view of the appraisal and coping process as a purely individual undertaking.

Reasoning from the perspective of the transactional model of stress (Lazarus and Folkman, 1984), the effects from team members’ coping efforts on a focal person’s outcomes are expected to result from influence on the appraisal process. In other words, the coping strategies used by team members may change the focal person’s appraisal of the situation, which in turn may influence his outcomes. Team members’ coping strategies may influence the focal person’s primary appraisal (making the situation seem less threatening), or the person’s secondary appraisal (making the situation seem more manageable). In both instances, the demanding situation will cause less stress, and thus result in less negative outcomes for the individual. Although our study does not provide a direct test of these effects on the appraisal process, our results do provide support for the idea that the appraisal and coping process deserves a broader perspective than merely the individual perspective.

Although the use of interpersonal strategies has always been part of theorizing about stress and coping (e.g., Van der Doef and Maes, 1999; Bakker et al., 2005), the present study shows that the influence of the social environment goes further; it is not just the social support one receives from others that helps in dealing with stress, it’s the way they cope with the stressors themselves that affects ones outcomes. To gain more insight in the effectiveness of coping strategies in settings where the exposure to demands is shared with others, it is therefore important to consider the influence of the team level as well. Ignoring team coping may obscure the influence of personal coping strategies, and limit the possibilities to draw sound conclusions. Disregarding the influence of the team level in our study, for example, would have led to the erroneous conclusion that the combined use of the strategies “active coping,” “positive reinterpretation,” “seeking social support,” “acceptance,” and “humor” is not effective for service members that have to deal with danger during deployments. Including team coping shows that using these strategies is, in fact, necessary for dealing with danger when the other team members do not use these strategies, and counterproductive when the other team members already do.

As such, inclusion of team coping as a higher-level construct in future coping research has the possibility to resolve some of the inconsistencies regarding the effectiveness of strategies found in previous research. Whereas it has been acknowledged that the same coping strategy may have a different effectiveness in different demand-outcome situations (e.g., Riolli and Savicki, 2010), the present findings suggest that the same strategy may even have a different effectiveness in similar demand–outcome situations, depending on the coping strategies used by team members in this situation.

### Practical Implications

Although further research into the concept of team coping is necessary, the present results already suggest some practical implications. Firstly, this knowledge may be used to better support professionals who have difficulties dealing with certain work-related stressors. Rather than focusing all attention on the struggling professionals, support could be organized more indirectly, by mobilizing coworkers who are better able to deal with the stressor. Stimulating interaction between the coworkers and these individuals might help them in better dealing with the stressor.

Furthermore, in high-risk professions (like the military), team coping should be considered as an additional resource for individual resilience at the team level, next to team resources such as unit cohesion, team work engagement, and team-efficacy (e.g., Kamphuis et al., 2012; Bates et al., 2013; Boermans et al., 2014), and attention should be paid to ways to facilitate the process of team coping. Currently, the focus in these professions is mainly on being able to deal with acute stressors in the moment. By stimulating contact between team members after these situations, and create possibilities to share coping experiences with each other, the positive effects of team coping could potentially be strengthened.

Finally, with a better understanding of how team coping exerts its positive influence and which behaviors play a role in this, leaders could be supported to stimulate these specific processes and behaviors in their teams. With knowledge about the mechanisms through which team coping influences individual outcomes, tailored approaches may be possible for different kinds of work-related demands and outcomes.

### Directions for Future Research

The present study confirmed that team coping influences individual outcomes. An important area for future research concerns the mechanisms by which the coping efforts of team members have the ability to influence the individual coping process. Two important topics are 1) the mechanism by which the coping efforts of coworkers are ‘transferred’ to and experienced by the individual, and 2) the way this experience influences the individual coping process. With regard to the first topic, future research needs to clarify whether the influence of coworkers primarily works in a conscious manner, through deliberate actions of talking about the experienced demands, or that the influence is more unconscious, comparable to processes like emotional contagion (e.g., Hatfield et al., 1993; Barsade, 2002), based on non-verbal cues and automatic processes. Furthermore, with regard to the second topic, future research should examine how the conscious or unconscious experience of the coworkers’ coping efforts influences the individual coping process. Does it affect the primary and/or secondary appraisal of the situation? Or does it more directly influence the coping behavior of the individual? In addition, future research should focus on the relationship between team coping (operationalized as the combined individual coping efforts to deal with common demands, influencing individual level outcomes) and coordinated strategies of the team as a whole to manage team task demands, aimed at maintaining team-level outcomes. Parallel to Bodenmann’s theory of dyadic coping (Bodenmann, 1997, 2005), different forms of coping in teams may be formulated, linked to outcomes at different levels (e.g., individual well-being and performance, team cohesion and performance). This may ultimately contribute to a better multi-level understanding of individual and team-level outcomes in demanding work environments.

### Limitations

A limitation of this study is the use of cross-sectional self-report data for all variables, introducing potential biases of common method and retrospective recall. However, because moderation effects cannot be inflated by common method variance (Siemsen et al., 2010), the three-way interaction found in this study is not an artifact of the method used. In addition, previous research with military personnel has shown that retrospective recall of events during deployments, such as exposure to danger, is not necessarily biased (Bramsen et al., 2001). Based on our design, however, we cannot rule out the possibility of alternative causal relations. Hypothetically, for example, it is possible that higher burnout led to more perceived danger, irrespective of the coping possibilities. Future research using longitudinal designs is necessary to confirm the direction of the effects and to determine whether the effects extend over time.

Our research approach did allow us to collect data from people in a naturalistic setting experiencing real stressors, contributing to the external validity of the results. Future research should determine, however, whether the findings from this study can be generalized to other organizational settings. Deployed military personnel operate in a unique work environment, with specific tasks and demands. Nonetheless, we expect that similar effects may be found in other work settings that rely on interdependent groups or teams of workers confronted with common work demands.

## Conclusion

To conclude, the present study has shown the relevance of treating coping as a multilevel construct and has demonstrated that including team coping in the model helps to explain individual-level outcomes. Based on these results, future coping research in work settings that rely on teams should account for the role of the team in explaining how work related demands influence individual outcomes. Including team coping will demonstrate in what other work settings the team plays a similar role in the coping process and will lead to more accurate models of stress and coping. Moreover, by specifically investigating the mechanisms that play a role in team coping, knowledge could be gained to better support employees in dealing with the demands they are confronted with as part of their work.

## Data Availability Statement

The datasets presented in this article are not readily available because the authors do not have approval from the Dutch Department of Defense to share data from military service members collected during deployments. Requests to access the datasets should be directed to wim.kamphuis@tno.nl.

## Ethics Statement

The studies involving human participants were reviewed and approved by the Internal Review Board, Behavioral Sciences Institute of the NLDAF. The patients/participants provided their written informed consent to participate in this study.

## Author Contributions

WK and RD formulated the research question, designed the study, and supervised data collection and analyses. WK wrote most of the introduction, the methods section, and the discussion. RD wrote a section of the introduction and reviewed other parts of the paper. TV performed the analyses and wrote the results section. All authors contributed to the article and approved the submitted version.

## Funding

This work was supported by the Knowledge and Innovation Department of the Dutch Ministry of Defense.

## Conflict of Interest

The authors declare that the research was conducted in the absence of any commercial or financial relationships that could be construed as a potential conflict of interest.

## Publisher’s Note

All claims expressed in this article are solely those of the authors and do not necessarily represent those of their affiliated organizations, or those of the publisher, the editors and the reviewers. Any product that may be evaluated in this article, or claim that may be made by its manufacturer, is not guaranteed or endorsed by the publisher.
